# A systematic review and meta-analysis of gender difference in epidemiology of HIV, hepatitis B, and hepatitis C infections in people with severe mental illness

**DOI:** 10.1186/s12991-018-0186-2

**Published:** 2018-05-04

**Authors:** Getinet Ayano, Mikiyas Tulu, Kibrom Haile, Dawit Assefa, Yodit Habtamu, Gebresilassie Araya, Zegeye Yohannis

**Affiliations:** Research and Training Department, Amanuel Mental Specialized Hospital, Addis Ababa, Ethiopia

**Keywords:** Epidemiology, HIV, Hepatitis B virus, Hepatitis C virus, Severe mental illness, Systematic review, Meta-analysis

## Abstract

**Background:**

People with severe mental disorders (SMDs) are associated with increased risk of infectious disease including human immunodeficiency virus infection (HIV) and hepatitis viruses, such as hepatitis B virus (HBV), hepatitis C virus (HCV), and other types of hepatitis viruses because of high-risk behaviors compared to the general population. The prevalence of HIV in people with SMDs is higher in females than in males. Unlike HIV, the prevalence of HBV and HCV is higher in males than in females. This study aimed to carry out a systematic review and meta-analysis to determine the prevalence and estimated gender difference in the risk of HIV, HBV, and HCV in people with SMD.

**Methods:**

Literature search was performed using the electronic databases PubMed, EMBASE, and Scopus. Publications were screened according to predefined inclusion criteria. A qualitative and quantitative analysis was undertaken for this systematic review. Eighteen materials published from 1993 to 2017 were included in the qualitative and quantitative analysis. Random-effect model was used to calculate weighted prevalence, odds ratio (OR), and corresponding 95% confidence interval (CI).

**Results:**

12,290 citations were identified and 18 articles including 11,175 participants were included. The results of our meta-analysis show that the prevalence of HIV, HBV, and HCV in people with SMD was 7.59% (95% CI 4.82–11.75), 15.63% (95% CI 7.19–30.69), and 7.21% (95% CI 4.44–11.50), respectively. The prevalence of HIV was higher in women (8.25%) than men (7.04%), but the prevalence of HBV and HCV was higher in men than women (18.91% versus 12.02% and 9.16% versus 5.43% for HBV and HCV in men versus women, respectively). A meta-analysis of included studies demonstrated a significantly increased risk of HBV (OR 1.72; 95% CI 1.17–2.53) and HCV (OR 2.01; 95% CI 1.16–3.20) infections in men compared to women in people with SMD. However, no significant association was observed between gender and HIV. The funnel plot and Egger’s regression tests provided no evidence of substantial publication bias in the prevalence and gender difference in association for HIV, HBV, and HCV in people with SMD.

**Conclusions:**

In our review, the prevalence of HIV, HBV, and HCV was high. The prevalence of HBV is significantly higher than HIV and HCV. There was a significantly increased risk of HBV and HCV infections in men compared to women. No significant association was observed between gender and HIV. People with SMDs warrant greater emphasis in efforts to identify and treat HIV, HBV and HCV.

**Electronic supplementary material:**

The online version of this article (10.1186/s12991-018-0186-2) contains supplementary material, which is available to authorized users.

## Background

People with severe mental disorders (SMDs), including schizophrenia, bipolar disorder and psychotic depression, are associated with increased risk of infectious disease including human immunodeficiency virus infection (HIV) and hepatitis viruses, such as hepatitis B virus (HBV), hepatitis C virus (HCV), and other types of hepatitis viruses because of high-risk behaviors compared to the general population [1–6].

A significant proportion of people with SMDs, are infected with HIV at some time in their lives with epidemiologically representative studies finding around 6.2–29.10% of people with SMDs had comorbid HIV infections [2, 7, 8]. The prevalence of hepatitis B and hepatitis C viruses in people with SMDs is significantly higher. According to different studies, the prevalence ranges from 7.45 to 47.5% [9–11] and 6.2–29.8% [2, 11, 12] for hepatitis B and hepatitis C, respectively.

The prevalence and risk of HIV, HBV, and HCV infections in people with SMDs differs by gender [8, 13, 14]. Studies indicated that the prevalence of HIV in people with SMDs is higher in females than in males. In one study, the prevalence of HIV was 33% in women and 19.7% in men [13]. Unlike HIV, the prevalence of hepatitis B and hepatitis C virus in people with SMDs is higher in males than in females. A study found that the rate of HCV infection among men was nearly twice that among women: 19.6% for male and 9.8% for female [14]. In another study, the prevalence of HBV was 12.6% for men and 7% for women [7]. A considerable gender differences in infection rates among people with SMDs might reflect differences in the patterns of risk behaviors as well as in the risk associated with a given behaviors.

Although it has been suggested that sex differences in the prevalence and risk of HIV, HBV, and HCV among people with SMDs exist, to date no systematic review or meta-analysis has examined this question. We therefore aimed to carry out a systematic review and meta-analysis to determine the prevalence and estimated gender difference in the risk of HIV, HBV, and HCV in people with SMDs.

## Methods/design

A systematic literature search was conducted on three databases, including EMBASE, PubMed, and Scopus. PubMed was searched using the following terms and keywords: epidemiology OR prevalence OR magnitude) AND (HIV OR human immune deficiency virus OR HBV OR HCV OR hepatitis OR AIDS) AND (severe mental illness OR mental disorder OR schizophrenia OR psychosis OR major depression OR bipolar disorder OR depressive disorder OR mental illness OR severe mental disorder). EMBASE and SCOPUS were searched as data-base specific subject headings (where applicable) associated with the above keywords used in PubMed. We scanned the reference lists of eligible studies to identify additional studies of relevance to this review. Preferred Reporting Items for Systematic Reviews and Meta-Analyses (PRISMA) guidelines, a checklist of 27 items that ensures the quality of systematic review or meta-analysis was used [15]. This review protocol was written and presented according to PRISMA-P 2015 guidelines [16].

### Eligibility criteria

Included in this systematic review and meta-analysis were studies that fulfill the following criteria. First, the study was done in people with SMDs; second, the study design was observational studies, including cross-sectional and case–control study design; third, the outcome of interest was infectious disease (HIV, HBV and HCV); fourth, the study reported the prevalence of HIV, HBV and HCV as well as risk in men and women. In additions, we excluded editorials, reviews, studies with nonhuman subjects, and those not published in English language. The identified studies were initially filtered with a title search by two reviewers before the retrieval of full-text articles for further screening. Rigorous inclusion criteria were adhered to. In the second step, the two reviewers independently read the full texts of the articles that were not excluded in the initial stage, then selected the studies that met the inclusion criteria. Disagreements were discussed during a consensus meeting with a third reviewer for final selection of studies to be included in the review. All differences of opinion regarding the selection of articles were resolved through discussion and consensus.

### Methods for data extraction and quality assessment

Data extraction from source documents was done independently by two investigators. Disagreements were resolved by consensus. The investigators used a specific form specifically designed to extract data of methodological and scientific quality. Data from the included papers were extracted to summary tables containing information on: population, study design, background information, sample size study setting, year of publication, authors, and tools used for assessing outcome and predisposing factors results. Information about design and participants was extracted as recommended by PRISMA (Preferred Reporting Items for Systematic Reviews and Meta-Analyses) guidelines [16].

A modified version of the Newcastle–Ottawa Scale was used to assess the quality studies included in our systematic reviews and meta-analyses [17]. This scale assesses quality in several domains: sample representativeness and size, comparability between participants, ascertainment of cases, and statistical quality. Moreover, the agreement between the two reviewers was assessed by actual agreement and by agreement beyond chance (unweighted Kappa) and these were interpreted as: # 0 = poor agreement, 0.01–0.20 = slight agreement, 0.21–0.40 = fair agreement, 0.41–0.60 = moderate agreement, 0.61–0.80 = substantial agreement, and 0.81–1.00 = almost perfect agreement [18].

### Data synthesis and analysis

Studies were pooled to calculate pooled prevalence, odds ratios, and 95% CIs using a random-effect model [19]. Comprehensive meta-analysis software version 3 was used for meta-analysis and forest plots that showed combined estimates with 95% CI. Heterogeneity was evaluated using *Q* statistic and the *I*^2^ statistics [19]. The magnitude of statistical heterogeneity between studies was assessed using *I*^2^ statistic and values of 25, 50, and 75% were considered to represent low, medium, and high, respectively [20]. For the data identified as heterogeneous, a random-effects model was used during analysis. When statistical pooling was not possible, non-pooled data were presented in table form. Meta-regression was performed to explore the potential source of heterogeneity. A leave-one-out sensitivity analysis was carried out to evaluate the key studies that exert major impact on between-study heterogeneity. Publication bias was assessed by funnel plot and Egger’s regression tests.

## Results

### Identification of studies

**Fig. 1:**
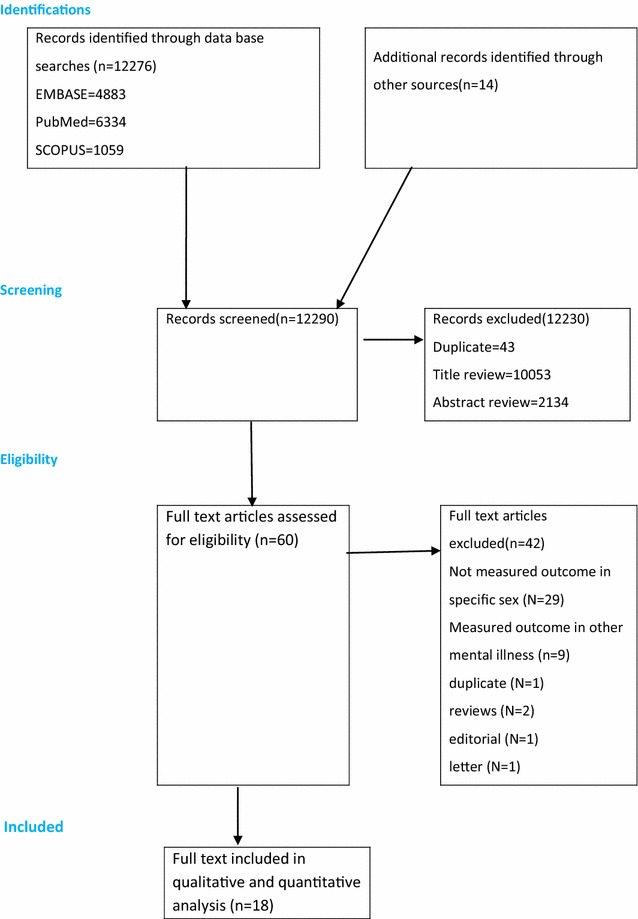
PRISMA flowchart of review search

The search identified 12,276 articles. An additional 14 relevant references were found through manual search of the reference lists of the remaining papers. Of these, 12,230 were excluded because of being duplicate and during the review of abstract and titles as they did not meet the inclusion criteria (Fig. 1). The full text of 60 articles was retrieved for further screening and 42 of these were excluded.

### Characteristics of included studies

The included studies had a total of 11,715 people with SMDs. The characteristics of these studies are summarized in Table 1. Selected studies were published between January 1993 [21] and July 2017 [22]. Nine studies were conducted in the USA [2, 8, 14, 21–26], two in Uganda [27, 28], and one in China [7], India [29], Lebanon [11], South Africa [13], Italy [10], Mexico [30], and Jordan [9].

**Table 1:** Characteristics of included studies

Author (year) (reference number)	Country	Sample size	Infectious disease	Prevalence
Klinkenberg et al. (2003) [2]	USA	172	HIV	Overall 6.2% (*n*/*N* = 11/172) Men 6.72% (*n*/*N* = 9/134) Women 5.26 (*n*/*N* = 2/38)
Hung et al. (2012) [7]	China	590	HBV and HCV	Overall HBV 10.4% (*n*/*N* = 59/590) HBV men 14.4% (*n*/*N* = 43/299) HBV women 7.50% (*n*/*N* = 16/212) Overall HCV 1.9% (*n*/*N* = 11/588) HCV men 2.6% (*n*/*N* = 9/337) HCV women 0.8% (*n*/*N* = 2/240)
Tharyan et al. (2003) [29]	India	1160	HIV	Overall 1.34% (*n*/*N* = 12/1160) Men 1.7 (11/663) Women 0.2 (1/497)
Singh et al. (2014) [8]	USA	206	HIV	Overall B29.1% (*n*/*N* = 60/206) Men 20.34 (*n*/*N* = 24/118) Women 40.1% (*n*/*N* = 36/88)
Kilbourne et al. (2004) [22]	USA	4310	HCV	Overall 5.9% (*n*/*N* = 252/4310) Men 6.23 (*n*/*N* = 242/3879) Women 2.33% (*n*/*N* = 10/431)
			HIV	Overall 0.8% (*n*/*N* = 35/4310) Men 0.88 (*n*/*N* = 34/3879) Women 0.23% (*n*/*N* = 1/431)
Siberstein et al. (2017) [22]	USA	117	HIV	Overall 23% (*n*/*N* = 27/117) Men 23.53% (*n*/*N* = 24/102) Women 20% (*n*/*N* = 3/15)
Cournos et al. (1991) [24]	USA	962	HIV	Overall 5.2% (*n*/*N* = 50/962) Men 5.2% (*n*/*N* = 29/563) Women 5.3% (*n*/*N* = 21/399)
Stanley et al. (2016) [11]	Lebanon	755	HIV	Overall 3% (*n*/*N* = 23/755) Men 3.1% (*n*/*N* = 16/512) Women 2.9% (*n*/*N* = 7/243)
			HBV and HCV	HCV Overall 14% (*n*/*N* = 109/755) Men 17.58 (*n*/*N* = 90/512) Women 7.8% (*n*/*N* = 19/243) Overall HBV 19% (*n*/*N* = 141/755)
Butterfield et al. (2003) [14]	USA	777	HCV	Overall 16.1% (*n*/*N* = 122/777) Men 19.8% (*n*/*N* = 98/526) Women 9.8% (*n*/*N* = 24/251)
Pamela et al. (2017) [13]	South Africa	151	HIV	Overall 26.5 (*n*/*N* = 40/151) Men 19.7% (*n*/*N* = 15/76) Women 33.3% (*n*/*N* = 25/75)
Maling et al. (2011) [27]	Uganda	622	HIV	Overall 18.4% (*n*/*N* = 50/272) Men 9.6% (*n*/*N* = 15/156) Women 30.2% (*n*/*N* = 35/116)
Lumberg et al. (2014) [28]	Uganda	602	HIV	Overall 11.3% (*n*/*N* = 68/602) Men 7.3% (*n*/*N* = 19/259) Women 14.3% (*n*/*N* = 49/343)
Nardo Di (1995) [10]	Italy	206	HBV and HCV	Overall HBV 47.5% (*n*/*N* = 97/206) HBV men 51.5% (*n*/*N* = 69/135) HBV women 39.44% (*n*/*N* = 28/71) Overall HCV 10.1% (*n*/*N* = 22/206) HCV men 8.9% (*n*/*N* = 12/135) HCV women 14.1% (*n*/*N* = 10/71)
Esquivel et al. (2005) [30]	Mexico	99	HBV	Overall 12.12% (*n*/*N* = 12/99) Men 13.04% (*n*/*N* = 9/69) Women 10% (*n*/*N* = 3/30)
Said et al. (2001) [9]	Jordan	188	HBV	Overall 7.45% (*n*/*N* = 14/188) Men 43% (*n/N* = 10/106) Women 4.88% (*n/N* = 4/82)
Empfield et al. (1993) [21]	USA	203	HIV	Overall 6.4% (*n*/*N* = 13/203) Men 6.5% (*n*/*N* = 10/146) Women 5.3% (*n*/*N* = 3/57)
Susser et al. (2015) [25]	USA	62	HIV	Men 19.4% (*n*/*N* = 12/62)
Stewart et al. (1994) [26]	USA	533	HIV	Overall 5.85% (*n*/*N* = 31/530) Men 5% (*n*/*N* = 17/339) Women 7.3% (*n*/*N* = 14/191)

### Quality assailment

All 18 studies were of good methodological quality. Reviewers agreed that the risk of selection, measurement, and non-response bias was low. Moderate or almost perfect agreement between reviewers regarding the level of bias was reached for all studies (Kappa statistic range 0.50–1) (Additional file 1).

### The results of pooled meta-analysis

#### The prevalence of HIV in people with severe mental illness

**Fig. 2:**
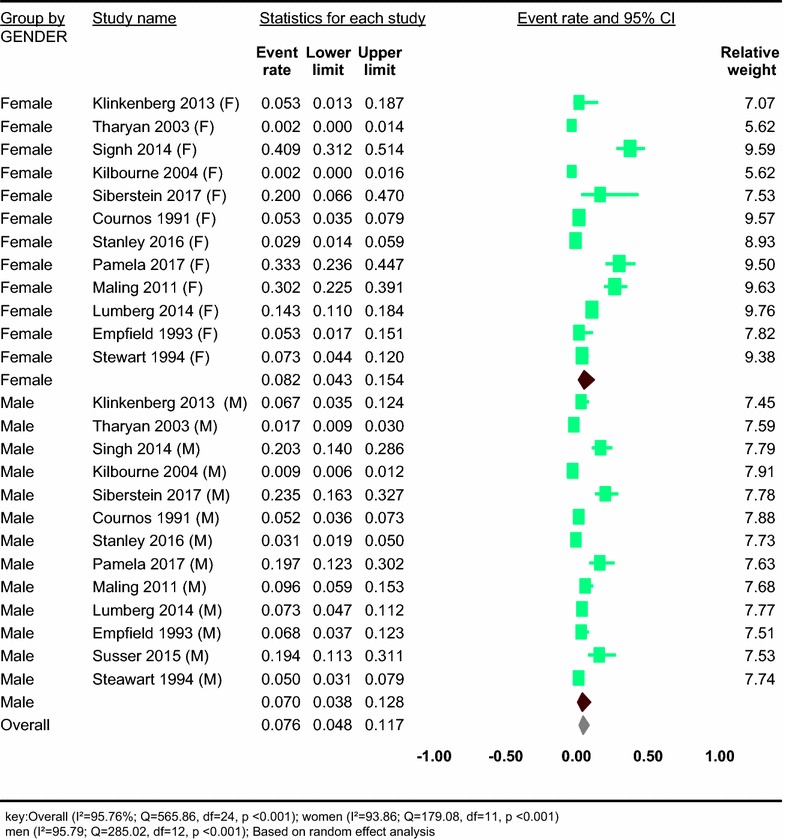
Forest plot of the prevalence of HIV in people with severe mental disorder: a meta-analysis

Thirteen studies reported on the prevalence of HIV in people with severe mental disorder-specific sex group (Table 1). Based on the results of the random-effects method, the pooled prevalence of HIV in people with SMDs was 7.59% (95% CI 4.82–11.75) and the heterogeneity was considerable (*I*^2^ = 95.76%; *Q* = 565.86, df = 24, *p* < 0.001) (see Fig. 2).

In our stratified analysis of 13 studies which reported the prevalence of HIV in people with SMDs in specific sex group, we found that the prevalence of HIV was higher in women 8.25% (95% CI 4.25–15.40) than men 7.04% (95% CI 3.75–12.82). A significant heterogeneity was found in both women (*I*^2^ = 93.86; *Q* = 179.08, df = 11, *p* < 0.001) and men (*I*^2^ = 95.79; *Q* = 285.02, df = 12, *p* < 0.001) (see Fig. 2).

#### Gender difference in the risk of HIV in people with severe mental disorder

**Fig. 3:**
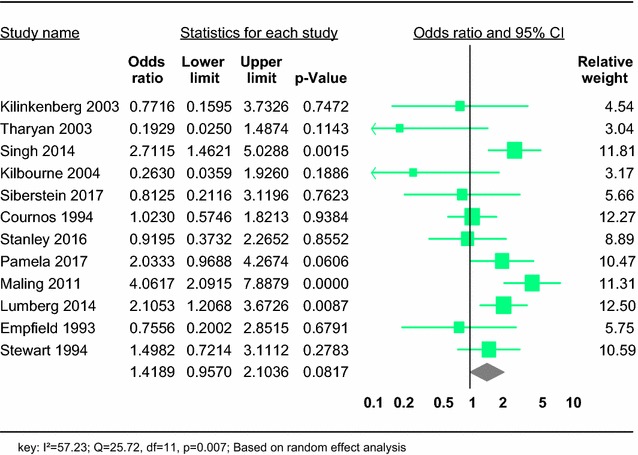
Forest plot of the risk of being female and HIV in people with severe mental disorder: a meta-analysis

We included 12 studies. Meta-analysis of crude odds ratio (OR) demonstrated no significant association between being female and HIV (OR 1.42; 95% CI 0.96–2.10) in people with SMDs (Fig. 3). We observed significant heterogeneity across the studies (*I*^2^ = 57.23; *Q* = 25.72, df = 11, *p* = 0.007).

#### The prevalence of hepatitis B virus in people with severe mental illness

**Fig. 4:**
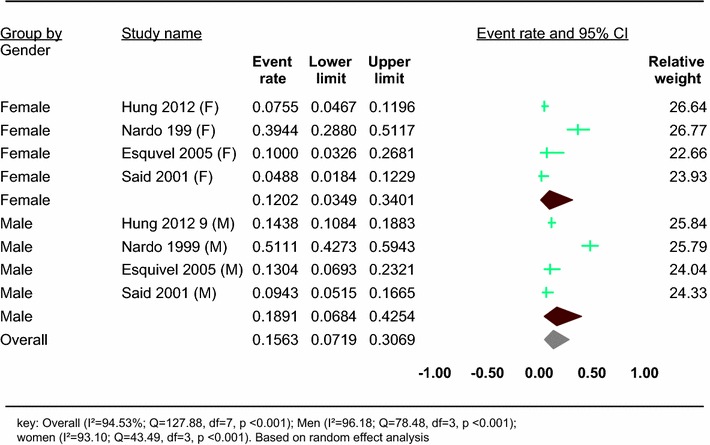
Forest plot of the prevalence of HBV in people with SMD: a meta-analysis

Four studies reported on the prevalence of HBV in people with SMDs in specific gender (Table 1). Based on the results of random-effects method, the pooled prevalence of HBV in people with SMDs was 15.63% (95% CI 7.19–30.69) and the heterogeneity was considerable (*I*^2^ = 94.53%; *Q* = 127.88, df = 7, *p* < 0.001) (Fig. 4).

In our subgroup analysis of four studies which reported the prevalence of HBV in people with SMDs in specific sex group, we found that the prevalence of HBV was higher in men, 18.91% (95% CI 6.84–42.54), than women, 12.02% (95% CI 3.49–34.01). A significant heterogeneity was found in both men (*I*^2^ = 96.18; *Q* = 78.48, df = 3, *p* < 0.001) and women (*I*^2^ = 93.10; *Q* = 43.49, df = 3, *p* < 0.001) (see Fig. 4).

#### Gender difference in the risk of hepatitis B virus in people with severe mental disorder

**Fig. 5:**
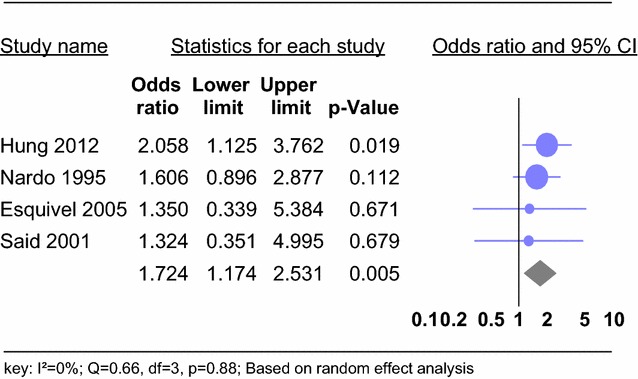
Forest plot of the risk of being male and HBV in people with SMD: a meta-analysis

We included four studies. Meta-analysis of crude odds ratio (OR) demonstrated a significant association between being male and HBV (OR 1.72; 95% CI 1.17–2.53) in people with SMDs (Fig. 5). No significant heterogeneity was observed across the studies (*I*^2^ = 0%; *Q* = 0.66, df = 3, *p* = 0.88).

#### The prevalence of hepatitis C virus in people with severe mental illness

**Fig. 6:**
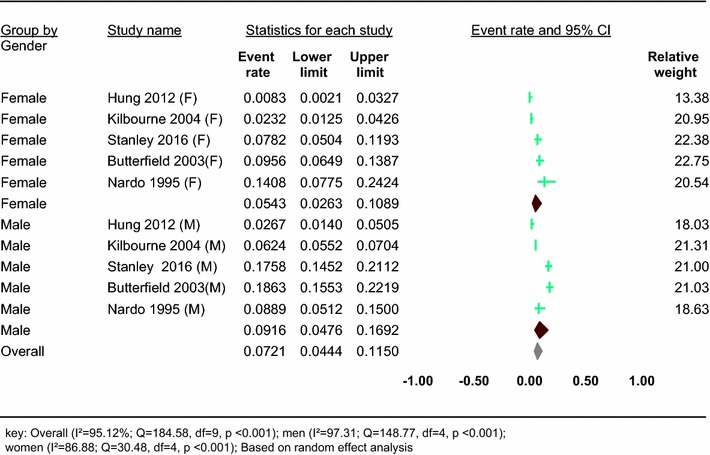
Forest plot of the prevalence of HCV in people with SMD: a meta-analysis

Five studies reported on the prevalence of HCV in people with SMDs in specific sex groups (Table 1). Based on the results of the random-effects method, the pooled prevalence of hepatitis C virus in people with severe mental disorder was 7.21% (95% CI 4.44–11.50) and the heterogeneity was considerable (*I*^2^ = 95.12%; *Q* = 184.58, df = 9, *p* < 0.001) (Fig. 6).

In our subgroup analysis of five studies which reported the prevalence of HCV in people with SMDs in specific sex groups, we found that the prevalence of HCV was higher in men, 9.16% (95% CI 4.76–16.92), than women, 5.43% (95% CI 2.63–10.89). A significant heterogeneity was found in both men (*I*^2^ = 97.31; *Q* = 148.77, df = 4, *p* < 0.001) and women (*I*^2^ = 86.88; *Q* = 30.48, df = 4, *p* < 0.001) (see Fig. 6).

#### Gender difference in the risk of hepatitis C virus in people with severe mental disorder

**Fig. 7:**
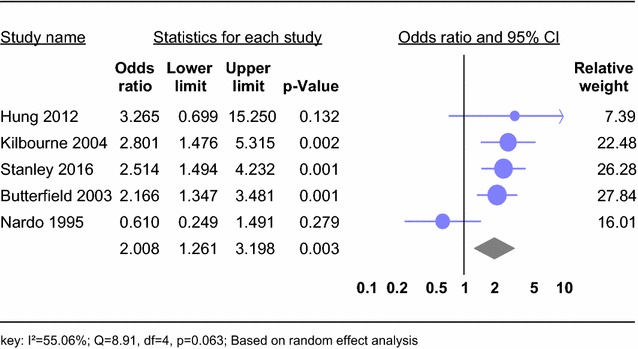
Forest plot of the risk of being male and HCV in people with SMD: a meta-analysis

We included five studies. Meta-analysis of crude odds ratio (OR) demonstrated a significant association between being male and HCV (OR 2.01; 95% CI 1.16–3.20) in people with SMDs (Fig. 7). No significant heterogeneity was observed across the studies (*I*^2^ = 55.06%; *Q* = 8.91, df = 4, *p* = 0.063).

### Publication bias

**Fig. 8:**
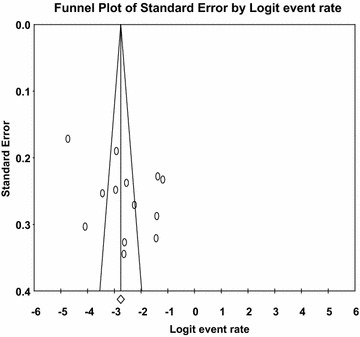
Funnel plot of publication bias for HIV in males with severe mental illness

**Fig. 9:**
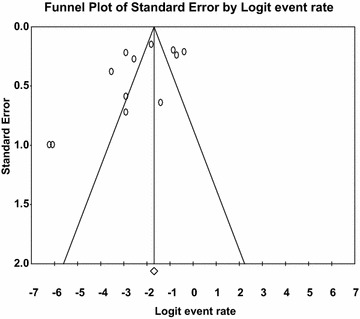
Funnel plot of publication bias for HIV in females with severe mental illness

The funnel plot was symmetric and Egger’s regression tests provided no evidence of substantial publication bias for the prevalence of HIV in people with SMD in males (*B* = 9.98, SE = 6.22, *p* = 0.137). However, the funnel plot was asymmetric for females, i.e., it showed the presence of small study effect, but Egger’s linear regression tests provided a two tailed non-significant *p* value (*B* = − 4.03, SE = 2.15, *p* = 0.09) (see Figs. 8 and 9). We did not find any evidence of publication bias for HBV and HCV due to the small number of studies in each gender category.

### Sensitivity and subgroup analysis

For the purpose of further investigating the potential source of heterogeneity in the analysis of the prevalence and gender difference in association with HIV, HBV, and HCV in people with SMDs, we performed leave-one-out sensitivity analysis to assess whether one study had a dominant effect on the summary of the study prevalence. Our sensitivity analysis showed that our findings were strong and not dependent on a single study. Our pooled estimated prevalence of HIV in people with SMD varied between 6.30% (3.37–11.51%) and 8.36% (5.20–14.55%) for males and 6.86% (3.27–13.03%) and 10.25% (5.35–18.55%) for females after deletion of a single study (see Additional file 2). In addition, our pooled estimated prevalence of HBV in people with SMD varied between 13.23% (3.97–16.61%) and 23.19% (10.45–56.80%) for males and 7.25% (2.76–10.67%) and 15.66% (5.35–48.17%) for females after deletion of a single study (see Additional file 3). Moreover, our pooled estimated prevalence of HCV in people with SMD varied between 7.50% (3.46–16.61%) and 11.84% (5.94–22.23%) for males and 4.17% (1.61–9.37%) and 7.18% (3.75–13.44%) for females after deletion of a single study (see Additional file 4).

When restricting the analysis to studies conducted to developed countries, the prevalence of HIV, HBV, and HCV was found to be 7.47, 19.37, and 6.2%, respectively, as compared to studies conducted in developing countries, 7.52, 7.53, and 12.10%, although the difference was not statistically significant (see Table 2). In addition, the prevalence of HIV, HBV, and HCV in developed countries in males was found to be 7.72, 23.19, and 7.61%, respectively, as compared to studies conducted in developing countries, 6.13, 9.43, and 17.58%, although the difference was not statistically significant except for the prevalence of HCV in men (see Table 2). Furthermore, the prevalence of HIV, HBV, and HCV in developed countries in females was found to be 7.11, 15.66, and 4.65%, respectively, as compared to studies conducted in developing countries, 9.63, 4.88, and 7.82%, though the difference was not statistically significant (see Table 2). Nevertheless, we found significant heterogeneity across the studies conducted in developed as well as developing countries with significant *p* value for heterogeneity for HIV, HBV, as well as HCV prevalence. Sufficient data were not found for performing stratified analysis by type of specific SMD including schizophrenia, psychotic depressive, bipolar, and schizoaffective disorders which we assumed to be the possible source of heterogeneity.

**Table 2:** Sensitivity analysis of all studies based on study quality and status of the country where the study was conducted

Subgroups	Studies, *n*	Prevalence (%)	95% CI	Type of infectious disease	Gender	Heterogeneity between groups (*P* value)
Country						0.989
Developed	15	7.47	3.79–14.28	HIV	Both	
Developing	10	7.52	3.75–14.51	HIV	Both	
Country						0.710
Developed	8	7.72	3.09–18.02	HIV	Men	
Developing	5	6.13	2.73–13.20	HIV	Men	
Country						0.660
Developed	7	7.11	242–19.09	HIV	Women	
Developing	5	9.63	2.73–22.70	HIV	Women	
Quality of studies						0.058
High	14	5.21	2.87–9.28	HIV	Both	
Moderate and poor	11	12.13	6.29–22.10	HIV	Both	
Quality of studies						0.352
High	7	6.43	2.96–13.39	HIV	Women	
Moderate and poor	5	12.03	3.95–31.24	HIV	Women	
Quality of studies						0.059
High	7	4.42	2.19–8.72	HIV	Men	
Moderate and poor	6	11.99	3.95–31.24	HIV	Men	
Country						0.059
Developed	3	19.37	8.84–37.32	HBV	Both	
Developing	1	7.53	4.03–13.62	HBV	Both	
Country						0.167
Developed	3	23.19	7.17–54.14	HBV	Men	
Developing	1	9.43	5.15–16.65	HBV	Men	
Country						0.172
Developed	3	15.66	3.79–46.68	HBV	Women	
Developing	1	4.88	1.84–12.29	HBV	Women	
Quality of studies						0.370
High	1	10.75	5.62–19.60	HBV	Both	
Moderate and poor	3	17.38	7.22–36.26	HBV	Both	
Quality of studies						0.609
High	1	14.38	10.84–18.83	HBV	Men	
Moderate and poor	3	20.57	4.85–56.80	HBV	Men	
Quality of studies						0.458
High	1	7.55	4.67–11.96	HBV	Women	
Moderate and poor	3	13.97	2.76–48.17	HBV	Women	
Country						0.182
Developed	4	6.21	3.56–10.62	HCV	Both	
Developing	1	12.10	5.29–25.35	HCV	Both	
Country						0.031
Developed	4	7.61	3.46–15.94	HCV	Men	
Developing	1	17.58	14.52–21.12	HCV	Men	
Country						0.352
Developed	4	4.65	1.65–12.41	HCV	Women	
Developing	1	7.82	5.04–11.93	HCV	Women	
Quality of studies						0.135
High	4	6.46	3.75–10.88	HCV	Both	
Poor	1	10.99	6.93–17.00	HCV	Both	
Quality of studies						0.940
High	4	9.20	4.33–18.49	HCV	Men	
Poor	1	8.89	5.12–15.00	HCV	Men	
Quality of studies						0.017
High	1	4.17	1.80–9.37	HCV	Women	
Poor	3	14.08	7.75–24.24	HBV	Women	

*HIV* human immunodeficiency virus infection, *HBV* hepatitis B virus, *HCV* hepatitis C virus

We further restricted the analysis to high-quality studies, and the prevalence of HIV, HBV, and HCV was found to be 5.21, 10.75, and 9.20%, respectively, as compared to moderate and poor-quality studies, 12.13, 17.38, and 8.89%, although the difference was not statistically significant (see Table 2). Moreover, the magnitude of HIV, HBV, and HCV differed based on the quality of studies for both males and females, but the difference was not statistically significant except for the prevalence of HCV in women (see Table 2).

## Discussion

In this study, we computed the pooled estimate of prevalence of infectious disease (HIV, HBV and HCV) as well as odds ratio (OR) of gender and infectious disease in people with SMDs. To our knowledge, this is the first systematic review and meta-analysis of gender difference in epidemiology of HIV, hepatitis B, and hepatitis C infections in people with SMDs. Based on the results from a meta-analysis, we identified a significant increase in risk and prevalence of hepatitis B and C viruses in men compared to women, but the prevalence of HIV in people with SMD was higher in women than in men.

The results of meta-analysis show that the pooled prevalence of HIV in people with SMDs was 7.59% (95% CI 4.82–11.75). Our finding of HIV was significantly higher than the 0.87, 0.00 (95% CI 0.00–0.003), and 0.6% of the general population in Niger [31], Iran [32], and the USA [5], respectively. The difference might be due to a significantly increased use of psychoactive substance and injective drugs as well as risky sexual behaviors in people with severe mental illness, which considerably increased the risk of having HIV infections.

In our stratified analysis of 13 studies which reported the prevalence of HIV in people with SMDs in a specific sex group, we found that the prevalence of HIV was higher in women, 8.25% (95% CI 4.25–15.40), than in men, 7.04% (95% CI 3.75–12.82). This difference might be because women with SMDs are more likely to experience violence, exploitation, abuse, or sexual assault than men. Nevertheless, in our meta-analysis of the risk of being female, the crude odds ratio (OR) demonstrated no significant association between being female and having HIV (OR 1.42; 95% CI 0.96–2.10) in people with SMDs.

The pooled results of our meta-analysis give the prevalence of hepatitis B virus in people with SMDs, 15.63% (95% CI 7.19–30.69). This prevalence was considerably higher than the general population prevalence of 0.4 and 0.9% in the USA [33] and Europe [6], respectively. The increased prevalence might be because people with SMDs are more likely to engage in risky sexual behavior including not using condom during sexual intercourse as well as an increased use of psychoactive substance and injective drugs, which are the major means of transmission of HBV.

In addition, in our study the pooled prevalence of HCV in people with SMDs r was 7.21% (95% CI 4.44–11.50). Our findings were significantly higher than the findings from the general population prevalence of 1.1% (95% CI 0.9–1.4) and 3.1% (95% CI 2.2–4.4) in Europe [6] and Ethiopia [34], respectively. The difference might be due to a considerably increased use of psychoactive substance and injective drugs as well as risky sexual behaviors in people with SMDs, which significantly increased the risk of having HCV infections.

Furthermore, the results of meta-analysis demonstrated a significantly increased risk of HBV (OR 1.72; 95% CI 1.17–2.53) and HCV (OR 2.01; 95% CI 1.16–3.20) infections in men compared to women in people with SMDs. The possible explanation might be due to men having significantly higher rates of lifetime substance and drug risks than women, including needle use, needle sharing, and crack cocaine use. Studies have indicated that people who inject drugs (PWID) are at risk for hepatitis B virus (HBV) and hepatitis C virus (HCV) infection through the sharing of needles and drug preparation equipment [35–37].

Unlike hepatitis virus, our meta-analysis of crude odds ratio (OR) demonstrated no significant association between being female and having HIV (OR 1.42; 95% CI 0.96–2.10) in people with SMDs. This might be because the rates of hepatitis virus transmission following needlestick injury are significantly higher than the rate of HIV transmissions through needles. In addition, hepatitis B virus can survive outside the body for at least 7 days, which might increase the risk of transmission [38, 39].

### Difference between studies

The difference between the 18 studies led to a high level of heterogeneity in our meta-analysis. The type of specific SMD, sample size, the setting, and the study populations differed on a number of characteristics, which will have contributed to the variance in the prevalence rates of HIV, HBV, and HCV in people with SMD. For the purpose of further investigating the potential source of heterogeneity in the analysis of the prevalence of HIV, HBV, and HCV, we performed leave-one-out sensitivity analysis. Our sensitivity analysis showed that our findings were strong and not dependent on a single study.

In addition, the robustness of our findings is indicated by our stratified analysis based on the quality of the included studies. The results were in line with our findings after removal of the poor quality studies [13, 22] for HIV [5.88% (3.61–9.45%)] and HBV [17.01% (6.58–37.36%)] for overall prevalence as well as 5.64% (2.97–10.46%) versus 6.23% (2.91–12.84%) for HIV and 21.11% (6.01–52.47%) versus 12.62% (2.76–42.30%) for HBV for men versus women, respectively.

Furthermore, for making the results of our meta-analysis meaningful, we used random-effects model where summary effect estimates are more conservative than fixed-effects summaries in epidemiologic meta-analysis.

## Strength and limitations

Our study has several strengths: First, we used predefined search strategy and data extraction, and quality assessment was performed by two independent reviewers to minimize the possible reviewer bias; Second, we performed sensitivity and subgroup analysis to identify the small study effect and the risk of heterogeneity. Third, we evaluated the quality of the included studies, and the result from the assessment of the study quality indicated that the methodological quality was generally good. However, we identified considerable heterogeneity among the studies which we considered as limitations of the current study.

## Conclusions

Results from this systematic review and meta-analysis suggest that: (1) the prevalence of HIV (7.59%), HBV (15.63%), and HCV (7.21%) was high; (2) the prevalence of HBV is significantly higher than HIV and HCV in people with SMDs; (3) there was a significantly increased risk of HBV and HCV infections in men compared to women; (4) there is no significant association between gender and HIV in people with SMDs; (5) prevention and routine screening of HIV, HBV, and HCV are warranted in people with SMDs; (6) the integrated management of SMDs and of HIV, HBV, and HCV is warranted; (7) psychiatry professionals should give attention to prevention, screening, and management of HIV, HBV, and HCV in people with SMDs; (8) further studies focusing on the incidence and outcomes of HIV, HBV, and HCV are recommended in people with SMDs; (9) finally, studies focusing on the reasons or factors related to significantly high prevalence and risk of HIV, HBV, and HCV in people with SMDs than the general population as well as the gender difference in association are warranted.

## Additional files

**Additional file 1.** Quality assessment of included studies.

**Additional file 2.** Sensitivity analysis of prevalence for each study being removed at a time: prevalence and 95% confidence interval of HIV in people with SMD.

**Additional file 3.** Sensitivity analysis of prevalence for each study being removed at a time: prevalence and 95% confidence interval of HBV in people with SMD.

**Additional file 4.** Sensitivity analysis of prevalence for each study being removed at a time: prevalence and 95% confidence interval of HCV in people with SMD.
