# Supplementary material for: A systematic review and meta-analysis of gender difference in epidemiology of HIV, hepatitis B, and hepatitis C infections in people with severe mental illness
Source: Ann Gen Psychiatry. 2018 May 4;17:16. doi: 10.1186/s12991-018-0186-2 (PMC5935990; doi:10.1186/s12991-018-0186-2)
Supplement: Supplementary file 1 — Additional file 1. Quality assessment of included studies. [file 12991_2018_186_MOESM1_ESM.docx]

**Additional file 1 :** Summary of agreed level of bias and level of agreement on the methodological qualities of included studies in meta-analysis based on sampling, outcome, response rate and method of analysis

| Study | Overall agreement and precision | | | Quality |
| --- | --- | --- | --- | --- |
|  | Percentage of agreement | Kappa value | Level of agreement |  |
| Klinkenberg WD. et.al(2003)[2] | 100 | 1 | Almost perfect | High |
| Hung CC. et. al (2012)[7] | 100 | 1 | Almost perfect | High |
| Tharyan P.et. al(2003)(27) | 75 | 0.60 | Moderate | Moderate |
| Singh D et.al (2014)(8) | 75 | 0.60 | Moderate | Moderate |
| Kilbourne AM.et. al(2004)(21) | 100 | 1 | Almost perfect | High |
| Siberstein C et. al(2017)(20) | 75 | 0.50 | Moderate | Poor |
| Cournos F.et. al(1991)(22) | 100 | 1 | Almost perfect | High |
| Stanley D.et. al(2016)(11) | 100 | 1 | Almost perfect | High |
| Butterfield MI .et.al(2003)(14) | 100 | 1 | Almost perfect | High |
| Pamela Y et.al(2017)(13) | 75 | 0.50 | Moderate | Poor |
| Maling S.et.al(2011)(25) | 100 | 1 | Almost perfect | High |
| Lumberg P.et.al(2014)(26) | 100 | 1 | Almost perfect | High |
| Nardo Di (1995)(10) | 75 | 0.60 | Moderate | Moderate |
| Esquivel CA.et.al(2005)(18) | 75 | 0.60 | Moderate | Moderate |
| Said WM .et.al(2001)(9) | 75 | 0.60 | Moderate | Moderate |
| Empfield M .et.al(1993)(29) | 75 | 0.60 | Moderate | Moderate |
| Susser E .et.al(2015)(23) | 75 | 0.60 | Moderate | Moderate |
| Stewart DL .et.al(1994)(24) | 100 | 1 | Almost perfect | High |
