# Supplementary material for: A systematic review and meta-analysis of gender difference in epidemiology of HIV, hepatitis B, and hepatitis C infections in people with severe mental illness
Source: Ann Gen Psychiatry. 2018 May 4;17:16. doi: 10.1186/s12991-018-0186-2 (PMC5935990; doi:10.1186/s12991-018-0186-2)
Supplement: Supplementary file 2 — Additional file 2. Sensitivity analysis of prevalence for each study being removed at a time: prevalence and 95% confidence interval of HIV in people with SMD. [file 12991_2018_186_MOESM2_ESM.docx]

**Additional file 2:** Sensitivity analysis of prevalence for each study being removed at a time: prevalence and 95% confidence interval of HIV in people with severe mental disorder by gender

| Study excluded | Gender | prevalence | 95%CI |
| --- | --- | --- | --- |
| Klinkenberg 2013 | Male | 7.07 | 3.62-13.36 |
|  | Female | 8.52 | 4.28-16.25 |
| Tharyan 2003 | Male | 7.89 | 4.13-14.55 |
|  | Female | 10.25 | 5.41-18.55 |
| Singh 2014 | Male | 6.40 | 3.37-11.82 |
|  | Female | 6.86 | 3.50-13.03 |
| Kilbourne 2004 | Male | 8.36 | 5.20-13.17 |
|  | Female | 10.16 | 5.35-18.45 |
| Siberstein 2017 | Male | 6.30 | 3.36-11.51 |
|  | Female | 7.60 | 3.77-14.73 |
| Cournos 1991 | Male | 7.23 | 3.58-14.07 |
|  | Female | 8.70 | 4.39-16.51 |
| Stanley 2016 | Male | 7.53 | 3.84-14.22 |
|  | Female | 9.25 | 4.76-16.71 |
| Pamela 2017 | Male | 6.43 | 3.35-11.99 |
|  | Female | 6.94 | 3.39-13.68 |
| Maling 2011 | Male | 6.86 | 3.49-13.06 |
|  | Female | 6.97 | 3.36-13.92 |
| Lumberg 2014 | Male | 7.02 | 3.53-13.47 |
|  | Female | 7.37 | 3.27-15.74 |
| Empfield 1993 | Male | 7.07 | 3.60-13.36 |
|  | Female | 8.55 | 4.28-16.34 |
| Steawart 1994 | Male | 7.24 | 3.66-13.85 |
|  | Female | 8.26 | 4.04-16.15 |
| Susser 2015 | Male | 6.45 | 3.35-12.05 |

Key. The analysis is based on random effect model
