# Supplementary material for: A systematic review and meta-analysis of gender difference in epidemiology of HIV, hepatitis B, and hepatitis C infections in people with severe mental illness
Source: Ann Gen Psychiatry. 2018 May 4;17:16. doi: 10.1186/s12991-018-0186-2 (PMC5935990; doi:10.1186/s12991-018-0186-2)
Supplement: Supplementary file 3 — Additional file 3. Sensitivity analysis of prevalence for each study being removed at a time: prevalence and 95% confidence interval of HBV in people with SMD. [file 12991_2018_186_MOESM3_ESM.docx]

**Additional file 3:** Sensitivity analysis of prevalence for each study being removed at a time: prevalence and 95% confidence interval of HBV in people with severe mental disorder by gender

| Study excluded | Gender | prevalence | 95%CI |
| --- | --- | --- | --- |
| Hung 2012 | Male | 20.57 | 4.85-56.80 |
|  | Female | 13.97 | 2.76-48.17 |
| Nardo 1999 | Male | 13.23 | 10.45-16.61 |
|  | Female | 7.25 | 4.86-10.67 |
| Esquivel 2005 | Male | 21.11 | 6.09-52.47 |
|  | Female | 12.61 | 2.76-42.30 |
| Said 2001 | Male | 23.19 | 3.97-46.68 |
|  | Female | 15.66 | 5.35-18.45 |

Key. The analysis is based on random effect model
