# Supplementary material for: A systematic review and meta-analysis of gender difference in epidemiology of HIV, hepatitis B, and hepatitis C infections in people with severe mental illness
Source: Ann Gen Psychiatry. 2018 May 4;17:16. doi: 10.1186/s12991-018-0186-2 (PMC5935990; doi:10.1186/s12991-018-0186-2)
Supplement: Supplementary file 4 — Additional file 4. Sensitivity analysis of prevalence for each study being removed at a time: prevalence and 95% confidence interval of HCV in people with SMD. [file 12991_2018_186_MOESM4_ESM.docx]

**Additional file 4:** Sensitivity analysis of prevalence for each study being removed at a time: prevalence and 95% confidence interval of HBV in people with severe mental disorder by gender

| Study excluded | Gender | prevalence | 95%CI |
| --- | --- | --- | --- |
| Hung 2012 | Male | 11.84 | 5.94-22.23 |
|  | Female | 7.18 | 3.71-13.44 |
| Kilbourne 2004 | Male | 10.41 | 5.79-18.00 |
|  | Female | 7.18 | 3.75-13.33 |
| Stanley 2016 | Male | 7.61 | 3.46-15.94 |
|  | Female | 4.65 | 1.65-12.41 |
| Butterfield 2003 | Male | 7.50 | 3.56-15.14 |
|  | Female | 4.40 | 1.61-11.47 |
| Nardo 1995 | Male | 9.20 | 4.33-18.49 |
|  | Female | 4.17 | 1.80-9.37 |

Key. The analysis is based on random effect model
